# Bearing Fault Diagnosis Based on Statistical Locally Linear Embedding

**DOI:** 10.3390/s150716225

**Published:** 2015-07-06

**Authors:** Xiang Wang, Yuan Zheng, Zhenzhou Zhao, Jinping Wang

**Affiliations:** 1 College of Water Conservancy and Hydropower Engineering, Hohai University, Nanjing 210098, China; E-Mail: wangjinping@njit.edu.cn; 2 School of Energy and Power Engineering, Nanjing Institute of Technology, Nanjing 211167, China; 3 College of Energy and Electrical Engineering, Hohai University, Nanjing 210098, China; E-Mails: zhengyuan@hhu.edu.cn (Y.Z.); zhaozhzh_2008@hhu.edu.cn (Z.Z.)

**Keywords:** high-dimensional data, fault diagnosis, feature extraction, dimensionality reduction, manifold learning, statistical locally linear embedding

## Abstract

Fault diagnosis is essentially a kind of pattern recognition. The measured signal samples usually distribute on nonlinear low-dimensional manifolds embedded in the high-dimensional signal space, so how to implement feature extraction, dimensionality reduction and improve recognition performance is a crucial task. In this paper a novel machinery fault diagnosis approach based on a statistical locally linear embedding (S-LLE) algorithm which is an extension of LLE by exploiting the fault class label information is proposed. The fault diagnosis approach first extracts the intrinsic manifold features from the high-dimensional feature vectors which are obtained from vibration signals that feature extraction by time-domain, frequency-domain and empirical mode decomposition (EMD), and then translates the complex mode space into a salient low-dimensional feature space by the manifold learning algorithm S-LLE, which outperforms other feature reduction methods such as PCA, LDA and LLE. Finally in the feature reduction space pattern classification and fault diagnosis by classifier are carried out easily and rapidly. Rolling bearing fault signals are used to validate the proposed fault diagnosis approach. The results indicate that the proposed approach obviously improves the classification performance of fault pattern recognition and outperforms the other traditional approaches.

## 1. Introduction

Bearing components play a critical role in rotating machinery, and their functionality is directly relevant to the operational performance, and consequently the reliability and safety of these machines and related systems. Therefore, it is essential to develop reliable condition monitoring and fault diagnosis methods to prevent roller bearings from malfunctioning. Vibration analysis is the most commonly used method for detecting roller bearing failures [1]. Nowadays, various fault diagnosis methods have been proposed for actual roller bearing fault detection based on vibration signals obtained from accelerometer sensors. 

Fault diagnosis of rolling bearings is now a very important research area in machinery engineering. The essence of fault diagnosis is pattern recognition and classification. Feature extraction is a critical part of pattern recognition. An optimal strategy for feature-based fault diagnosis is to find a feature extraction technique extracting the most salient features beneficial to classification and simultaneously decreasing feature dimensionality [2], so more effective feature extraction methods and more accurate classifiers are needed to obtain higher diagnostic accuracy.

A challenging problem of fault diagnosis is how to deal with the high-dimensional and nonlinear data collected from the complete information of operating machinery. A large amount of data provides more available information, while also increasing the problem of effectively using these data, as the useful knowledge might be submerged in a large number of redundant data which increases the feature extraction difficulty. The approach to the problem is to apply dimensionality reduction to the data for the object of learning and classification. The purpose of dimensionality reduction is to obtain a more compact representation of the original high-dimensional data, a representation that nonetheless captures all the information necessary for higher-level decision-making. For fault feature extraction, the classical dimensionality reduction methods include principal component analysis (PCA) [3], multi-dimensional scaling (MDS) [4] and linear discriminate analysis (LDA) [5]. However, these approaches are only effective on datasets with a linear structure and a Gaussian distribution. It is difficult for us to use these methods to discover nonlinear structure in the fault data, resulting, from the point of view of fault classification, in low accuracy fault identification or misjudgment. Among traditional nonlinear mapping methods, Sammon mapping [6] and the neuroscale method [7] are used. The former uses an iterative process that results in intensive computation, while the latter uses a radial basis function network and has similar shortcomings as neural networks. Meanwhile, manifold learning, a new, effective nonlinear dimensionality reduction method, has attracted more and more attention recently. The approach provides a new means for intelligent fault diagnosis.

Compared with linear methods, the purpose of manifold learning methods is to project the original high-dimensional data into a lower dimensional feature space by preserving the local neighborhood structure, and they are effective for us to discover the intrinsic structure of nonlinear high-dimensional data for data analysis. At present, the representative methods include isometric mapping (Isomap) [8], locally linear embedding (LLE) [9], Laplacian Eigenmaps (LapEig) [10], local tangent space alignment (LTSA) [11], *etc.* Today, manifold learning methods are widely used in cluster analysis, image processing, bioinformatics, *etc.* For instance, the LLE algorithm is used to extract characteristic MR features of brain alterations [12] and solve face recognition problems [13]. A local embedding method based on LLE and a semi-supervised LapEig algorithm is presented to achieve lower dimensionality from high-dimensional data and implement data visualization and classification [14,15]. Furthermore, manifold learning is relatively seldom studied in the fault diagnosis field. Yang [16] proposed a nonlinear time series noise reduction method based on principal manifold learning applied to the analysis of gearbox vibration signals with snaggletooth, which was only for signal denoising. The LTSA algorithm as a typical manifold learning method used for dimensionality reduction from original high-dimensional feature datasets [1]. However, this traditional manifold learning is an unsupervised learning method, and cannot be applied efficiently to supervised learning problems.

In some fault diagnosis tasks, data are from multiple classes and the class label information is known, which can help in classification tasks. The information provided by these class labels may be used to guide the dimensionality reduction procedure.

For the supervised expansion of manifold learning, a supervised LLE method (SLLE) for classification problems by utilizing the class label information was proposed by Ridder *et al.* [17]. Although SLLE improves the performance of LLE related to classification, the information provided by the Euclidean distance between samples is not sufficient to select proper neighbors for classification. A supervised manifold learning approach based on correntropy LLE with class labels information for visualization and classification on noisy artificial and real-world datasets was proposed by Genaro *et al.* [18]. Based on probability-based distance and the supervised locally linear embedding technique, a novel dimension reduction method for classification is introduced in [19]. A novel supervised manifold learning technique called Supervised Laplacian Eigenmaps (S-LE) was proposed by Raducanu *et al.* [20], which makes use of class label information to guide the non-linear dimensionality reduction procedure for face recognition problems by adopting the large margin concept. Zhang *et al.* introduced in [21] a supervised feature extraction method called locally discriminating projection (LDP) and achieved good recognition accuracy. A new supervised manifold learning algorithm based on the S-LapEig algorithm for machinery fault diagnosis was introduced in [22] by Jiang *et al.*, and the good performance of the approach on a series of benchmark and real fault datasets verified its feasibility and efficiency. Su *et al.* [23] proposed a fault diagnosis method based on supervised extended local tangent space alignment (SE-LTSA) for dimension reduction to improve the effectiveness of fault diagnosis in machinery. Most of these methods are based on the improvement of manifold learning methods and to solve a certain task.

In this paper, aimed at addressing the difficulty of handling high-dimensional nonlinear fault data, we propose a new fault classification approach based on supervised manifold learning for rolling bearing fault diagnosis. Because of the prominent properties of considering both the local geometry information and the class information of the data, the proposed approach has efficient capability to deal with the supervised learning problem. Some experiments with the proposed method show its feasibility and effectiveness.

The remainder of the paper is organized as following: in Section 2, LLE theory and manifold learning methods are reviewed, and a new supervised manifold learning (statistical locally linear embedding, S-LLE) algorithm for feature extraction or reduction is proposed. The implementation steps of the algorithm are described in detail. In Section 3, we discuss a rolling bearing fault diagnosis strategy, and introduce the implementation process and flow chart of the proposed approach. In Section 4, we illustrate a feature extraction method by utilizing time-domain, frequency-domain and EMD analysis of the original rolling bearing vibration fault signals. In Section 5, we introduce the rolling bearing fault experiment setup and signal acquisition first, and then implement feature dimension reduction experimentally by utilizing S-LLE, and comparisons with other feature reduction methods such as PCA, LDA and LLE are also discussed. Finally, the proposed fault diagnosis approach is applied to pattern classification experiments with the original data and reduction of statistical features extracted from multi-domain rolling bearing vibration fault signals by three classification algorithms, namely, CART, K-NN and RBF-SVM, and the classification performance is analyzed and discussed. Finally, the conclusions are presented in Section 6. 

## 2. Statistical Locally Linear Embedding Algorithm

### 2.1. Locally Linear Embedding Algorithm

The locally linear embedding (LLE) algorithm was originally proposed by Roweis *et al.* [9] to achieve non-linear dimension reduction. LLE is an unsupervised learning algorithm that can compute low-dimensional features that preserve the neighborhood relationship as that of the high-dimensional data. In other words, the low dimensional space is required to preserve the neighborhood configuration. The LLE algorithm to compute a lower-dimensional representation of the original data is described as follows.

**Figure 1:**
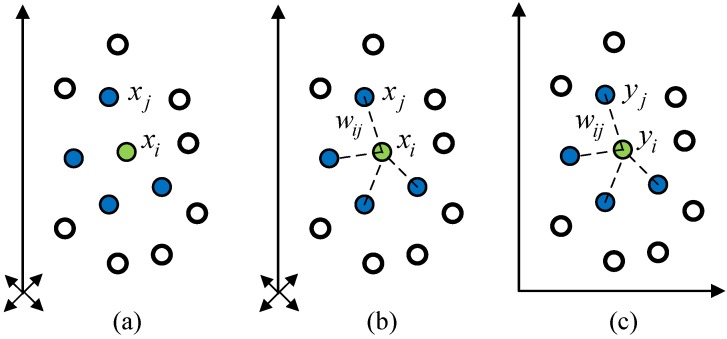
Illustration of LLE algorithm: (**a**) Select neighbors; (**b**) Reconstruct with embedded linear weights; (**c**) Map to coordinates.

Given a set of data, $X=\left\{x_{1},x_{2},\cdots x_{n}\right\}$ is in a high-dimensional input data space $R^{D}$. The data points are assumed to lie on or near a nonlinear manifold of intrinsic dimensionality d<D. The goal of LLE is to find a low-dimensional embedding of dataset X by mapping the *D*-dimensional data into a single global coordinate system in Euclidean distance $R^{D}$. The LLE algorithm can be generalized to three steps: select neighbors, reconstruct with linear weights and map to embedded coordinates. The steps of the LLE algorithm are illustrated in Figure 1.

*Step 1*: Using the Euclidean distance to define neighborhood of each input vector. Consider the i-th sample $x_{i}$ with K-nearest neighbors $x_{j}$ (i≠j). The neighborhood of each sample provides prior knowledge for LLE and affects the reconstruction result. The nearest neighbors of each sample can be identified by selecting a fixed number K of nearest neighbors in the Euclidean distance. Another approach is to choose samples within a fixed radius r as neighbors. The process of neighborhood selection can be flexible and various. 

*Step 2*: Reconstructing each sample by the linear combination of its neighbors. Compute the best reconstruct weights $w_{ij}$ of each input sample, $w_{ij}>0$ and $\sum_{j}w_{ij}=1$. The optimal reconstruction weight matrix $W_{n\times n}$ can be derived from minimizing the reconstruction error by properly selecting the reconstruction weights. The reconstruction error is thus formulated as:

(1) $$\text{ε}(W)=\sum_{i=1}^{n}{\left\|x_{i}-\sum_{j=1}^{n}w_{ij}x_{j}\right\|}^{2}$$

*Step 3*: Computing the coordinates of the original high dimensional data $x_{i}$ in the low dimensional space. The low dimensional embedding is obtained based on the idea that LLE preserves the local linearity from neighbors and the corresponding reconstruction weights $W_{n\times n}$. Using $W_{n\times n}$, the low dimensional output space Y can be computed by minimizing the cost function:

(2) $$\Phi (Y)={\left\|y_{i}-\sum_{j=1}^{n}w_{ij}y_{j}\right\|}^{2}$$

Subject to two constraints: $\sum_{i=1}^{N}y_{i}y_{i}^{T}/N=I_{d\times d}$, $\sum_{i=1}^{N}y_{i}=0$, where $w_{ij}$ is the same linear combination weight in the high-dimensional space and where Y is an n×d embedding data matrix (being d≤D), and $y_{i}\in R^{d}$ is the output sample vector, and 0 is a column vector of zeros. Based on W, we can define a sparse, symmetric, and positive semi definite matrix M as follows:

(3) $$M={\left(I-W\right)}^{T}(I-W)$$

Then, rewriting Equation (3) to find Y:

(4) $$\Phi (Y)=tr(Y^{T}MY)\;s.t.\{\begin{array}{l} I_{1\times n}Y=0_{1\times d}\\ Y^{T}Y/n=I_{d\times d} \end{array}$$

It is possible to calculate d + 1 eigenvectors of M, which are associated to the d + 1 smallest eigenvalues. The first eigenvector is the unit vector with all equal components, which is discarded. The remaining d eigenvectors constitute the embedding coordinates found by LLE. 

### 2.2. Statistical Locally Linear Embedding Algorithm

As mentioned above, the class label information is not utilized in the LLE dimensionality reduction process. The statistical locally linear embedding algorithm (S-LLE) is proposed based on utilizing class label information in the LLE algorithm to improve performance. The main idea of S-LLE is to utilize sample statistics to measure the relationship between samples. The membership of a sample laying on a nonlinear manifold can still be estimated using either parametric or nonparametric approaches. 

In the parametric approach, each class is decomposed to a set of clusters (subclasses) because the high dimensionality data is complicated and cannot be modeled by only one Gaussian model. A cluster-space representation for classification is extended to estimate the pairwise similarity between samples [24]. The *K*-means clustering algorithm [25] was selected due to its efficiency and easy implementation. The purpose of the *K*-means algorithm is to partition data into a fixed number of K clusters by minimizing the mean squared error distance. After clustering, the training sample would be labeled into KL clusters, where L is the number of classes and K is the number of clusters of each class. That means we have KL subclasses. The probability density of a sample X with D dimension in subclass ${\text{ω}}_{ik}$ can be defined as:

(5) $$p(x|\omega_{ik})=\frac{1}{{\left(2\pi \right)}^{D/2}{\left|{S^{\prime }}_{ik}\right|}^{1/2}}\exp\left\{-\frac{1}{2}{\left(x-m_{ik}\right)}^{T}{\left({S^{\prime }}_{ik}\right)}^{-1}(x-m_{ik})\right\}$$

where ${\text{ω}}_{ik}$ is the *k*-th cluster in class i, $m_{ik}$ is the mean of and ${S^{\prime }}_{ik}=S_{ik}+r\cdot I$ is the sample covariance matrix of ${\text{ω}}_{ik}$ plus a small value r times identity matrix to prevent singularity. 

Since the subclasses are mutually exclusive and statistically exhaustive, the likelihood of sample x in a class can be determined by the sum overall the subclasses, is given by:

(6) $$p(x|\omega_{i})=\sum_{k=1}^{K}\frac{n_{ik}}{n_{i}}p(x|\omega_{ik})$$

where $n_{i}$ is the number of samples of the *k*-th class, and n is the number of samples of *k*-th cluster in class i.

In the nonparametric approach, the Parzen-window density estimation technique [26] was used to estimate the membership of samples and devise the statistic based similarity between samples. The Parzen-window defined a D-dimensional hypercube local region V with its length h to estimate density, *i.e.*,$V=h^{D}$, the number $k_{n}$ falls into the hypercube V centered at x given by:

(7) $$k_{n}=\sum_{i=1}^{n}\text{φ}(\frac{x-x_{i}}{h})$$

where φ(⋅) is a kernel function defined as:

(8) $$\text{φ}(x)=\{\begin{array}{c} 1,\\ 0, \end{array}\begin{array}{c} \left\|x\right\|<1/2\\ otherwise \end{array}$$

Therefore the density can be estimated as:

(9) $$p(x|\omega_{j})=\frac{1}{n_{j}}\sum_{i=1}^{n}\frac{1}{V}\text{φ}(\frac{x-x_{i}}{h})$$

where $n_{j}$ is number of samples in class ${\text{ω}}_{j}$. Thus the likelihood of a sample for each class can be estimated as follows:

(10) $$p(x|\omega_{i})=\sum_{k=1}^{K}p(x|\omega_{ik})$$

Without loss of generality, we assume that samples are independent. Therefore, the likelihood of samples x and $x^{\prime }$ belonging to the same class ${\text{ω}}_{i}$ is equal to the product of the individual likelihoods. A new distance measure between any pair of samples can be defined as:

(11) $$d(x,x^{\prime })=\underset{i}{\max}\left\{-\log p(x|\omega_{i})p(x^{\prime }|\omega_{i})\right\}$$

The measurement d incorporates the class information provided by the statistical cluster model for each class. In S-LLE, d is used to find the neighbors of each sample instead of the original Euclidean distance in the first step of LLE algorithm. Later, the *K*-nearest neighbors of each sample are determined according to d. The subsequent steps follow the same procedure as LLE.

Both the parametric and nonparametric approaches can be used in statistical LLE to estimate the class-conditional probability density function of samples. The statistical LLE algorithm improves the performance of LLE in classification applications by incorporating class label information. 

## 3. Statistical Locally Linear Embedding Algorithm for Bearing Fault Diagnosis

In this paper, we propose a new nonlinear dimensionality reduction method based on supervised manifold learning theory, which is a new fault diagnosis approach called the S-LLE algorithm. Taking special consideration of both the information of labeled data and local neighbor geometry information, the algorithm can obtain the whole intrinsic geometry of the dataset, and has good data classification performance. The approach first learns the intrinsic geometric structure of the fault data in the signal space to capture the nonlinear embedded manifold features and map the high-dimensional fault data into a low-dimensional embedded space. The process of the presented algorithm consists of two parts: training and testing. First we divide the fault dataset into a training dataset and a test dataset. Using the proposed S-LLE algorithm, the class label information is used to determine neighbors of the training dataset so as to map overlapping high-dimensional data into clusters in the embedded space, we map the training dataset into a low-dimensional feature space, and exploit the characteristic patterns of the dataset.

**Figure 2:**
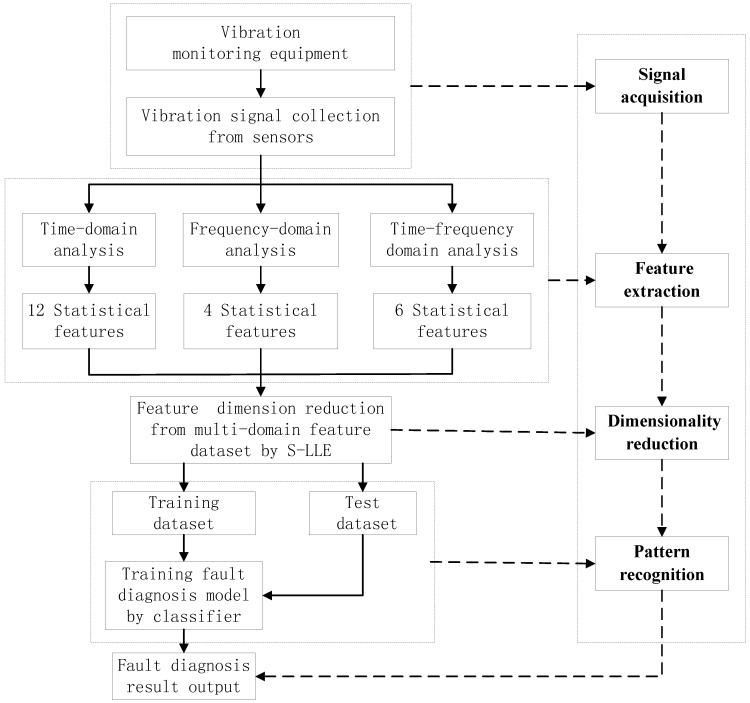
The implementation process and flow chart of the proposed approach.

In order to further improve the diagnosis performance and ensure the diagnosis reliability, the rolling bearing fault diagnosis model based on the supervised manifold learning S-LLE approach can be described in four main steps as follows: signal acquisition, feature extraction, dimensionality reduction and pattern recognition. The implementation process and flow chart of the proposed approach is shown in Figure 2:

(1) Signal acquisition: The acquisition of the original vibration signals is the first step in the rolling bearing fault diagnosis process.
(2) Feature extraction: Feature extraction directly characterizes the information relevant to the bearing conditions and greatly affects the final diagnosis results. The time-domain, frequency-domain and time–frequency domain features extracted from the original vibration signal by the empirical mode decomposition method are utilized to construct the multi-domain fault feature dataset.
(3) Dimensionality reduction: The multi-domain feature set can fully represent the bearing faults. However, all of these high-dimensional feature vectors are not independent of each other and there is much redundant information embedded in the high-dimensional feature space. In addition, different features have different importance in the different fault states. In order to reduce the computation time for the diagnosis model, the supervised manifold learning method S-LLE is used to select the salient features from the raw statistical feature dataset.
(4) Pattern recognition: Implementing fault classification of the training samples in the low-dimensional embedded space according to class label information and learning geometric structure feature by optimized classifiers. To test the dataset, we also map it onto the same feature space according to the mapping matrix of the training dataset, and evaluate the classification capability. Finally, pattern recognition is carried out in the embedded spaces. In order to reliably diagnose complex roller bearing faults, the proposed fault diagnosis approach is applied for the roller bearings fault diagnosis.

Compared with other fault diagnosis methods, this method has several advantages as follows:

(1) The method is based on nonlinear dimensionality reduction and can treat high-dimensional nonlinear data, which avoids the “curse of dimensionality”.
(2) The method can capture more accurately the intrinsic geometric distribution properties of samples by the sample label information, and utilize the obtained distribution feature to classify the fault category.
(3) The feature extraction method based on time-domain, frequency-domain and time-frequency domain is simple and the implementation speed is high, which greatly reduces the fault diagnosis difficulties.

## 4. High-Dimensional Fault Features Extracted from Accelerometer Sensor Vibration Signals

In recent years, intelligent fault diagnosis based on statistical features has received extensive attention because it can exploit important fault-related information contained in machinery operating vibration signals, and many fault diagnosis methods are proposed based on the features extracted from vibration signals [27]. Vibration signal analysis is an important means in the field of online detection and fault diagnosis of mechanical equipment. Generally, these signals are generated by accelerometer sensors on rolling bearings [28]. When faults occur in a rolling bearing, the vibration signal in this malfunction condition will deviate in the time-domain from that of normal condition both in amplitude and phase position. Meanwhile, the amplitude and distribution in the frequency spectrum of the transformed vibration signal also will change under different fault conditions [1]. 

The essential aim of signal processing is to map a signal from the time domain into another space in which some important information of the signals can be revealed, and consequently, some dominant features of the signals can be extracted [29]. When faults or abnormal running states occur, the measured vibration signals are usually non-stationary and non-linear, and the components of the vibration signals are also very complicated. Time-frequency domain methods are considered to be best way for analyzing the nonlinear and non-stationary signals of faulty bearings [30,31] due to the deficiencies of the Fourier transform. Wavelet transform [32,33] is a commonly used time-frequency domain signal analysis method, but the results of wavelet transform are related to the selected wavelet basis. Empirical Mode Decomposition (EMD) [30] doesn’t need a base function and is completely based on the local characteristic time scale of the signal, so EMD is a self-adaptive signal processing method that is applicable to non-stationary and non-linear vibration signals and fault diagnosis of rotating machinery [34,35].

Many research results show that a multi-domain feature set can fully represent the bearing fault feature information which can provide an effective diagnosis for various faults of rolling bearings operating under variable speed and load or unknown speed conditions. For this purpose, various original features that can be extracted from accelerometer sensor signals of bearings have been investigated. This section presents a vibration signal feature calculation method from the time-domain, frequency-domain, and time-frequency domain as they will be used in a high-dimensional fault feature dataset.

A large set of statistical feature parameters has been defined in the process of roller bearing fault diagnosis, among which, six dimensional time-domain features including mean value ($T_{m}$), root mean square ($T_{rms}$), root value ($T_{r}$) standard deviation ($T_{sd}$), skewness ($T_{sk}$), Kurtosis ($T_{ku}$) and six dimensionless time-domain features including shape indicator ($T_{sf}$), crest factor ($T_{cf}$),impulse factor ($T_{if}$), clearance factor ($T_{clf}$), skewness factor ($T_{skf}$), kurtosis factor ($T_{kuf}$) and four frequency-domain statistical parameters including mean frequency ($F_{mf}$), frequency center ($F_{fc}$), root mean square frequency ($F_{rmsf}$) and root variance frequency ($F_{rvf}$) are used to construct the roller bearing statistical feature set as shown in Table 1 and Table 2. These statistical features have demonstrated their effectiveness in previous publications, not only independent of speeds and loads, but to some extent they also can indicate complex roller bearing faults.

**Table 1:** Time-domain features.

No.	Dimensional Features	Feature Definition	No.	Dimensionless Features	Feature Definition
1	Mean	$T_{m}=\frac{1}{n}\sum_{i=1}^{n}x_{i}$	7	Shape factor	$T_{sf}=\frac{T_{rms}}{\bar{x}}$
2	Root mean square	$T_{rms}={\left[\frac{1}{n}\sum_{i=1}^{n}x_{i}^{2}\right]}^{1/2}$	8	Crest factor	$T_{cf}=\frac{x_{\max}}{x_{rms}}$
3	Root	$T_{r}={\left[\frac{1}{n}\sum_{i=1}^{n}{\left|x_{i}\right|}^{1/2}\right]}^{2}$	9	Impulse factor	$T_{if}=\frac{x_{\max}}{\bar{x}}$
4	Standard deviation	$T_{sd}={\left[\frac{1}{n-1}\sum_{i=1}^{n}{\left(x_{i}-\bar{x}\right)}^{2}\right]}^{1/2}$	10	Clearance factor	$T_{clf}=\frac{x_{\max}}{x_{r}}$
5	Skewness	$T_{sk}=\frac{\sum_{i=1}^{n}{\left(x_{i}-\bar{x}\right)}^{3}}{(n-1)T_{sd}^{3}}$	11	Skewness factor	$T_{skf}=\frac{T_{sk}}{T_{rms}^{3}}$
6	Kurtosis	$T_{ku}=\frac{\sum_{i=1}^{n}{\left(x_{i}-\bar{x}\right)}^{4}}{(n-1)T_{sd}^{4}}$	12	Kurtosis factor	$x_{kuf}=\frac{T_{ku}}{T_{rms}^{4}}$

Here $x_{i}$ is a signal time series for i=1,2,...,n, and n is the number of data points, $x_{\max}=\max\left|x_{i}\right|$ and $\bar{x}=\frac{1}{n}\sum_{i=1}^{n}\left|x_{i}\right|$ is the absolute mean value.

**Table 2:** Frequency-domain features.

No.	Features	Feature Definition	No.	Features	Feature Definition
1	Mean frequency	$F_{mf}\text{=}\sum_{i=1}^{N}p_{i}/N$	3	Frequency center	$F_{fc}=\frac{\sum_{i=1}^{N}f_{i}p_{i}}{\sum_{i=1}^{N}p_{i}}$
2	Root mean square frequency	$F_{rmsf}={\left(\frac{\sum_{i=1}^{N}{f_{i}}^{2}p_{i}}{\sum_{i=1}^{N}p_{i}}\right)}^{\frac{1}{2}}$	4	Root variance frequency	$F_{rvf}={\left(\frac{\sum_{i=1}^{N}{\left(f_{i}-F_{mf}\right)}^{2}p_{i}}{\sum_{i=1}^{N}p_{i}}\right)}^{\frac{1}{2}}$

Here $p_{i}$ is the power spectrum of x(i), i=1,2,...,N, N is the number of spectrum liness, and $f_{i}$ is the frequency value of the i-th- spectrum line. $F_{mf}$ can indicate the vibration energy in the frequency domain, $F_{fc}$ and $F_{rmsf}$ describe the position change of the main frequency, and $F_{rvf}$ may show the centralized or decentralized degree of the spectrum power energy.

However, when the roller bearing fault is at an early stage, it is very hard to distinguish the feeble fault features only from the time-domain and frequency-domain signals. In this study, we adopt EMD energy to further mine for more characteristic information for accurate roller bearing diagnosis. EMD energy can reveal the original vibration signal in the time-frequency amplitude and distribution view. Some previous researches show that the typical roller bearing faults can be identified by EMD energy [30,34].

The original vibration signal is decomposed into a finite stationary intrinsic mode function (IMF) by the EMD method, the procedure needed to generate the adaptive IMF basis. An IMF is defined as any function satisfying the following conditions:

(1) In the whole dataset, the number of extrema and the number of zero crossings must either equal or differ by at most one;
(2) At any point, the mean value of the envelope defined by the local maxima and the envelope defined by the local minima is zero.

Based on EMD algorithm, the rolling bearing original vibration signal x(t) can be decomposed into a set of IMFs:

(12) $$x(t)=\sum_{j=1}^{n}c_{j}(t)+r_{n}(t)$$

where $c_{j}(t)$ is the j-th- IMF of the signal x(t) which represents different frequency bands ranging from high frequency to low frequency, and $r_{n}(t)$ is the final residue which can be a monotonic trend. More detailed information about EMD can be found in [5].

For an arbitrary time series x(t), we can always have its Hilbert transform y(t):

(13) $$y(t)=\frac{1}{\pi }p\int_{-\infty }^{\infty }\frac{x(\tau )}{t-\tau }d\tau$$

where *P* represents the Cauchy principal value. With this definition x(t) and y(t), we can have an analytic signal z(t):

(14) $$z(t)=x(t)+iy(t)=a(t)e^{i\theta (t)}$$

In which, $a(t)={\left[x{\left(t\right)}^{2}+y{\left(t\right)}^{2}\right]}^{1/2}$, $\text{θ}(t)=\arctan\frac{y(t)}{x(t)}$ It is also well know that the amplitude of the analytic signal a(t), in Equation (13) represents the envelope signal x(t). The time derivative of the phase θ(t) will be the instantaneous frequency of the signal ω(t), as $\text{ω}(t)=\frac{d\theta (t)}{dt}$. Therefore, the IMF component $c_{j}(t)$ can be expressed in the following representation as follows:

(15) $$c_{j}(t)=a_{j}(t)e^{i\int \omega_{j}(t)dt}$$

where $a_{j}(t)$ is the amplitude of *the* j-th IMF component using Hilbert transform. The amplitude energy of each IMF is computed as follows:

(16) $$E_{i}=\sum_{m=1}^{N}a_{j}(t_{m})$$

where m=1,2,...,N, *N* denotes the discrete data length of j-th IMF and $t_{m}$ is the amplitude of discrete point m in *the* j-th IMF component. The time-frequency domain feature vector set I with the amplitude energy is constructed as follows, and I is regulated by normalizing the feature for the convenience of the following diagnosis:

(17) I={E1,E2,...,En}

where n is the IMF number. In this study, the first six intrinsic mode functions (IMFs) containing almost all the valid information are selected. When different faults occur in the roller bearing, each IMF component will change in terms of its intrinsic information. By an energy calculation for each IMF component, the characteristic information of the original signal can be extracted more fully and effectively. Thus, the whole multi-domain feature set containing 22 statistical features is constructed, which is composed of 12 time domain, four frequency domain and six time-frequency domain terms.

## 5. Roller Bearing Fault Diagnosis Experiments and Analysis

### 5.1. Experiment Setup and Signal Acquisition

**Figure 3:**
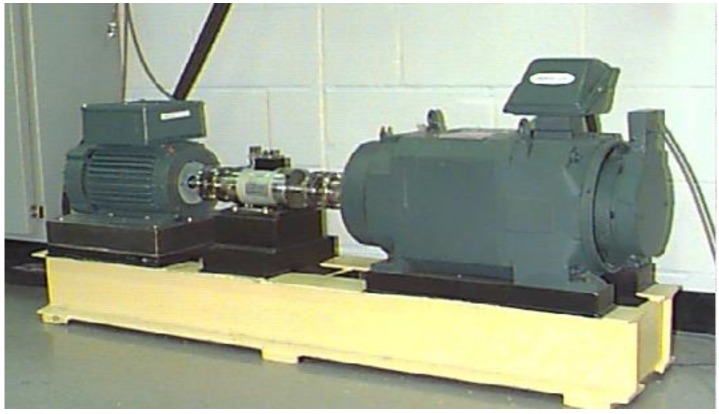
The rolling bearing fault test-bed.

To evaluate the effectiveness of the feature extraction and dimensionality reduction methods for rolling bearings, bearing vibration data of different faults related to the bearing in this paper were provided by the Bearing Data Center of the Case Western Reserve University (CWRU), and acquired by bearing accelerometer sensors under different operating loads and bearing conditions [36]. The bearing data have been validated in many researches [37,38] and has become a standard dataset for rolling bearings. In this experiment, the rolling bearings are installed in a motor-driven mechanical system. As shown in Figure 3, the rolling bearing fault test rig is made up of a 2 HP motor (left), a torque transducer/encoder (center), and a dynamometer (right). 

**Figure 4:**
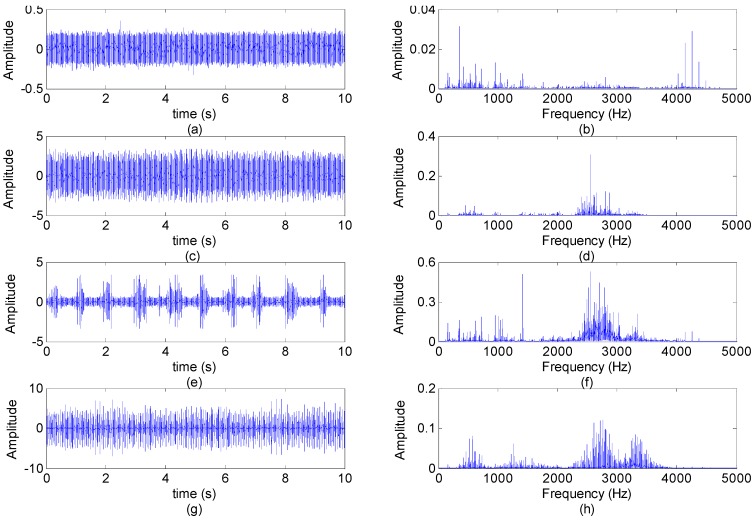
The vibration signal waveforms and power spectra from the different fault types: (**a**,**b**) Normal bearing vibration waveform/power spectrum; (**c**,**d**) Inner race fault vibration waveform/power spectrum; (**e**,**f**) Ball fault vibration waveform/power spectrum; (**g**,**h**) Outer race fault vibration waveform/power spectrum.

The bearing type is SKF6205-2RS JEM, a deep groove ball bearing. Four types of vibration signal datasets (normal, ball fault, inner race fault and outer race fault) are acquired from the bearings with the sampling frequency of 48 kHz during about 10 s by using a 16 channel DAT recorder, and tested under motor loads is 2 HP at the speed of 1750 r/min. A single point fault is introduced to the test bearing inner race and outer race, respectively, using an electro-discharge machining with the fault diameter of 21 mils inches and the fault depth of 11 mils (1 mil = 25.4 um). More detailed information about the test rig can be found in [36]. The length of the signal data in every dataset is about 480,000, we can extract 100 samples for each vibration condition, that is, every sample data includes 4096 points, and thus the overall dataset consists of 400 samples. Figure 4 presents the vibration signal waveforms and power spectra from four signal samples of the different fault types. 

### 5.2. Feature Extraction

**Figure 5:**
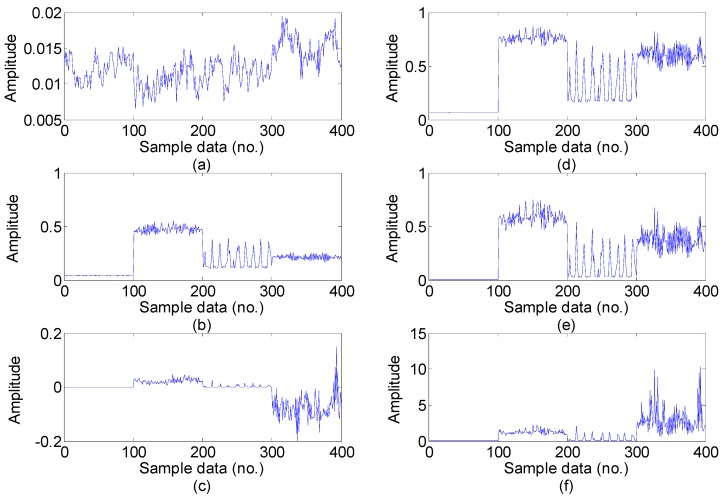
The six dimensional time-domain features value in the dataset: (**a**) Mean; (**b**) Root mean square; (**c**) Root; (**d**) Standard deviation; (**e**) Skewness; (**f**) Kurtosis (Note: sample data No.1–100, 101–200, 201–300, 301–400, represent normal, inner race fault, ball fault and outer race faults, respectively).

**Figure 6:**
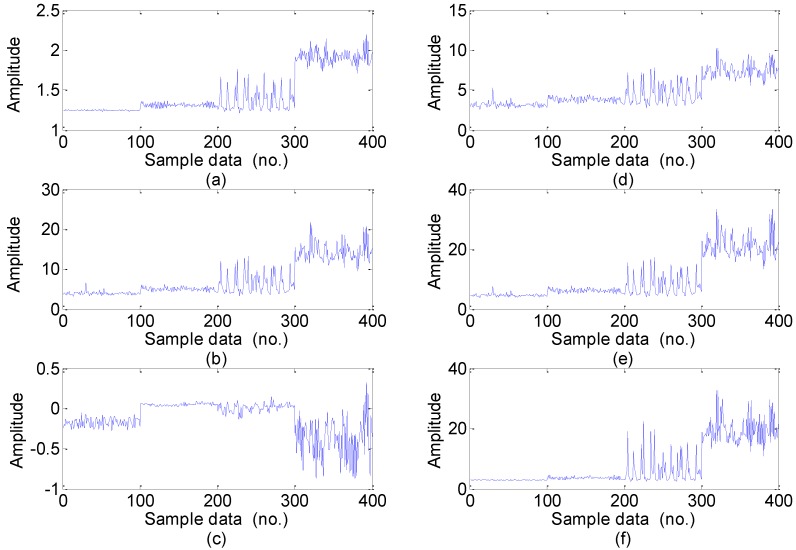
The six dimensionless time-domain features value in the dataset: (**a**) Shape factor. (**b**) Crest factor; (**c**) Impulse factor; (**d**) Clearance factor; (**e**) Skewness factor; (**f**) Kurtosis factor (Note: sample data No.1–100, 101–200, 201–300, 301–400 represent normal, inner race fault, ball fault and outer race faults, respectively).

**Figure 7:**
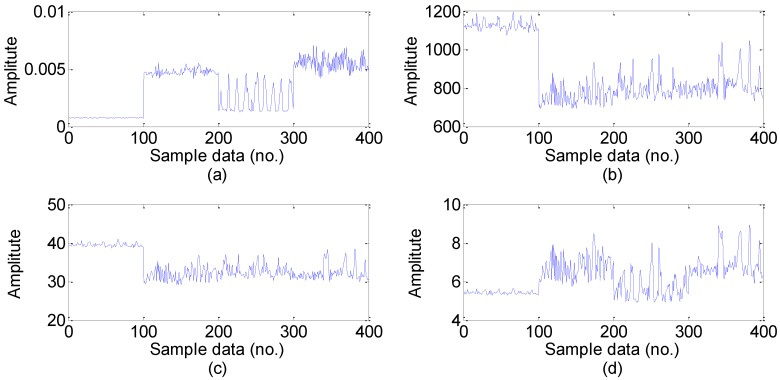
The four frequency-domain features value in the dataset: (**a**) Mean frequency; (**b**) Frequency center; (**c**) Root mean square frequency. (**d**) Root variance frequency (Note: sample data No.1–100, 101–200, 201–300, 301–400 represent normal, inner race fault, ball fault and outer race faults, respectively).

**Figure 8:**
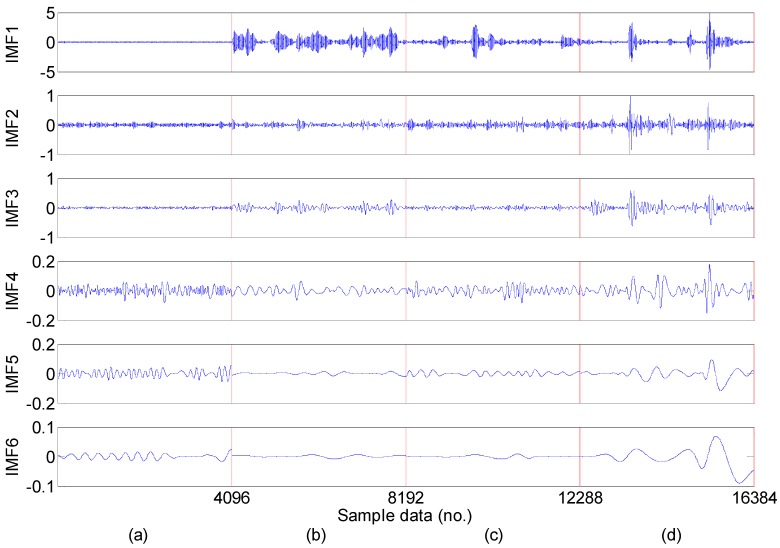
The first six IMFs obtained by applying EMD method to a signal sample in the dataset: (**a**) Normal; (**b**) Inner race fault; (**c**) Ball fault; (**d**) Inner race fault (Note: sample data No. 1–4096, 4097–8192, 8193–12288, 12289–16384 represent normal, inner race fault, ball fault and outer race faults, respectively).

For the every obtained data set, we extract statistical 22 features following the time-domain, frequency domain and time-frequency domain for the next feature dimension reduction. Twelve time-domain and four frequency-domain statistical features could be calculated directly using the feature definition equations as shown in Table 1 and Table 2, and time-frequency domain features are extracted from the EMD energy. The calculated value of the six dimensional and six dimensionless time-domain statistical features are shown in Figure 5 and Figure 6, respectively, and the calculated value of the four frequency-domain statistical features are shown in Figure 7. The six time-frequency domain statistical features obtained from the first six IMFs energy are obtained by applying EMD method. The EMD result of a signal sample in the dataset is shown in Figure 8.

**Figure 9:**
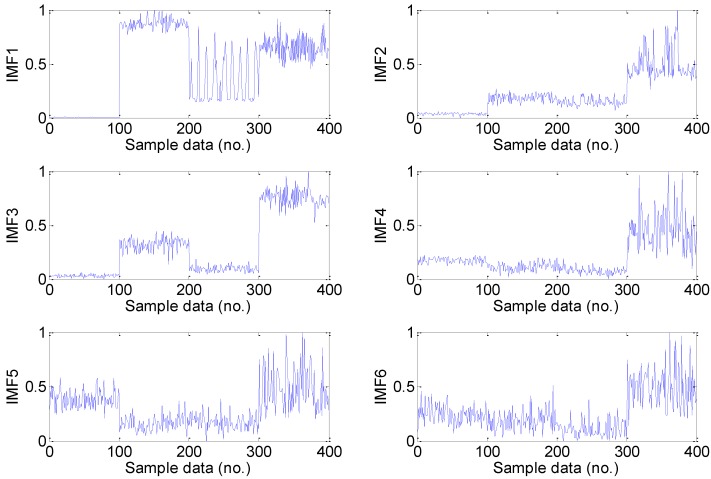
The normalized amplitude energy features value of the first six IMFs by EMD method (Note: sample data No. 1–100, 101–200, 201–300, 301–400 represent normal, inner race fault, ball fault and outer race faults, respectively).

The normalized IMFs energy was analyzed after EMD, and the results are shown in Figure 9, and the energy distributions are mutually different. From Figure 9, we note that there is a relatively large difference between the normal bearing and the faulty bearing signals.

### 5.3. Feature Dimension Reduction

In traditional diagnosis methods, these 22 features value are calculated from vibration signals to construct feature vectors which are directly input into the diagnosis model for rolling bearing fault diagnosis. However, due to the fact the diagnosis model involves too many parameters, directly applying a model on such high-dimensional feature set is very complicated and inefficient. Based on the manifold learning theory, the specific samples in the same state have the same distribution property and geometric structure in the sample space. The samples in different states also have different embedded manifolds. One only needs to select the salient features by a manifold learning algorithm to recover the geometric distribution embedded in the high-dimensional fault features [1].

**Figure 10:**
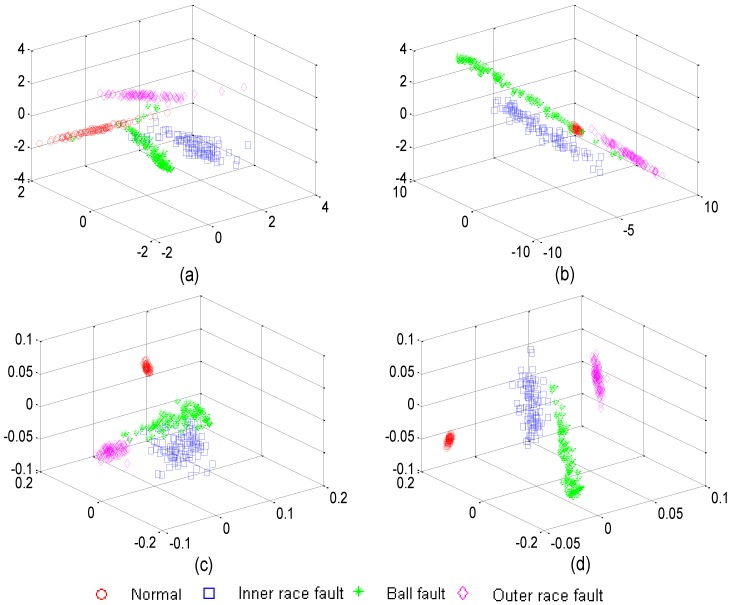
Feature dimension reduction to rolling bearing multi-domain feature in the dataset: (**a**) Mapping with PCA; (**b**) Mapping with LDA; (**c**) Mapping with LLE; (**d**) Mapping with S-LLE.

In order to demonstrate the superiority of the presented S-LLE dimensionality reduction method, when S-LLE is carried out in the process of the training sample labeled into KL clusters, K is set to 4 and L is set to 4. An experiment was conducted on the dataset to evaluate its dimensionality reduction performance on the sample dataset and make a comparison with PCA, LDA, and LLE as the most representative dimensionality reduction approaches. The experimental results of dimensionality reduction with the four approaches are shown in Figure 10, where it can be seen that PCA, LDA and LLE have poor sample classification performance. PCA and LDA obviously have three classes of overlap and LLE obviously has two classes of overlap. Compared with them, S-LLE can obtain a more clear separation of the clustering on the mapping, so S-LLE can identify each fault accurately for all feature samples. This is due to the fact that S-LLE has a greater ability to discovery local neighbor geometry information in the data manifold by utilizing the class label information. Therefore, we can use the S-LLE algorithm to obtain the original multi-domain feature dataset and select the salient features. This added process can capture intrinsic global geometric structure embedded in the high-dimensional fault features and achieve an efficient classification for fault pattern recognition.

### 5.4. Classification Performance Analysis

In this section, three classifiers are adopted to evaluate the performance of the feature reduction method based on S-LLE, which are classification and regression trees (CART), K-nearest-neighbor classifier (K-NN, K = 4), and support vector machine with RBF kernel (RBF-SVM). In this study, PRTools [39] is used to implement K-NN, and CART classifier, and LIBSVM [40] is used to implement the SVM classifier. For the RBF-SVM classifier, there is a parameter C to control the trade-off between the margin and the size of the slack variables, and there is also a parameter γ for the RBF kernel function. Hence, we will use the ten-fold cross-validation and the gird search to find the best C within the given set {$2^{-5},2^{-3},\cdots ,2^{15}$} and the best γ within the given set {$2^{-15},2^{-13},\cdots ,2^{3}$} of parameters to optimize RBF-SVM classifier.

**Figure 11:**
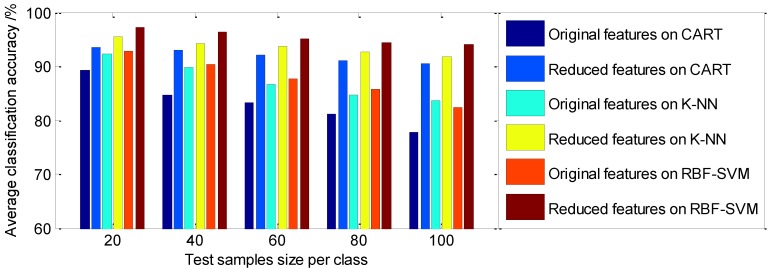
The comparison of the average classification accuracy with different features dataset on classifiers using statistical LLE.

These classifiers trained on the reduced feature dataset are compared to that of classifiers trained on the 22 original statistical feature dataset. In this experiment, 50 signal samples per class are selected randomly as a training dataset, thus 200 samples are collected as the training dataset to calculate the fitness function and construct the diagnosis model, and 20, 40, 60, 80, 100 samples per class, respectively, are selected as the test dataset to test the classification accuracy rate. Each experiment is carried out ten times, to give ten classification results. The average classification accuracy using statistical LLE is presented in Table 3. Figure 11 shows the visualized representation of the comparison result of the average classification accuracy between the proposed method and the classical methods. 

**Table 3:** The average classification accuracy (%) the original and reduction of statistical features extracted from multi-domain by various classifiers using statistical LLE.

Test Samples Size per Class	CART		K-NN		RBF-SVM	
	Original Feature	Reduced Feature	Original Feature	Reduced Feature	Original Feature	Reduced Feature
20	89.24	93.56	92.35	95.43	92.79	97.26
40	84.63	93.05	89.87	94.25	90.35	96.34
60	83.35	92.17	86.61	93.78	87.63	95.22
80	81.14	91.10	84.75	92.63	85.81	94.35
100	77.76	90.54	83.56	91.84	82.32	94.07

**Figure 12:**
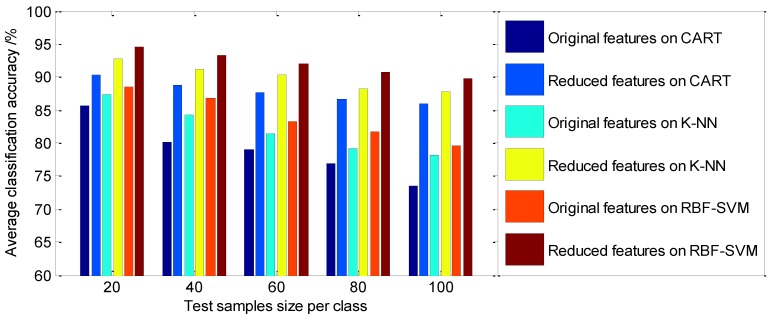
The comparison of the average classification accuracy with different features dataset on classifiers using supervised LLE.

Further comparison on the classification effect between statistical LLE and supervised LLE [17] methods which all utilize the class label information, the parameter α which controls the amount to which class label information fully used should be incorporated is set to 0.5, using the same calculation procedure as above, the average classification accuracy using supervised LLE is presented in Table 4, and the visualized representation is shown in Figure 12.

**Table 4:** The average classification accuracy (%) the original and reduction of statistical features extracted from multi-domain by various classifiers using supervised LLE.

Test Samples Size per Class	CART		K-NN		RBF-SVM	
	Original Feature	Reduced Feature	Original Feature	Reduced Feature	Original Feature	Reduced Feature
20	85.72	90.34	87.38	92.67	88.45	94.53
40	80.12	88.75	84.26	91.19	86.73	93.34
60	78.95	87.63	81.47	90.26	83.24	92.06
80	76.84	86.58	79.15	88.23	81.76	90.69
100	73.45	85.93	78.22	87.84	79.57	89.78

As shown in Table 3 and Table 4 and Figure 11 and Figure 12, it can be seen that various classifiers perform better on the reduced feature dataset than on the original statistical feature dataset, and the average classification accuracy by various classifiers using the statistical LLE method is higher than that using supervised LLE. From Table 3, among these classifiers, the CART classifier works the worst on the original statistical feature set (the average classification accuracy is in the range of 77.76%–89.24%), whereas the reduced features followed by the CART classifier perform relatively well (the average classification accuracy is in the range of 90.54%–93.56%). Among the three classifiers, the optimized RBF-kernel SVM has a higher diagnostic accuracy than that of CART, and K-NN in roller bearing fault diagnosis, and the accuracies on the reduced feature dataset are in the range of 94.07%–97.26%, and on the original feature dataset they range from 82.32% to 92.79%. Therefore, a reasonable feature dimension reduction method is a necessary step prior to final classification on account of the fact the original feature dataset contains too much fault unrelated or redundant information. Overall, from the above experimental results, it can be seen that the parameter-optimized SVM model has high diagnostic accuracy; these experimental results also indicate that the proposed diagnosis model is obviously superior to the traditional diagnosis methods with reduced feature datasets. This demonstrates the effectiveness of S-LLE for feature dimensionality reduction of the given input space, and also confirmed the obviously improveed performance of the classifier. 

## 6. Conclusions

In this paper, aimed at addressing the difficulty of dealing with high-dimensional nonlinear fault data, we propose a new fault reduction and classification approach based on statistical locally linear embedding (S-LLE) for rolling bearing fault diagnosis. Because of the prominent features of considering both the local geometry information and the class information of the data, the proposed approach efficiently deals with the supervised learning problem. For dealing with the test fault samples, the approach applies S-LLE to find the projection that best approximates the implicit mapping from high-dimensional feature samples dataset to their embedding. The experimental result show that S-LLE outperforms the other traditional dimensionality reduction methods such as PCA, LDA and LLE. Finally fault classification is carried out in the embedded space. Some experiments show the RBF-SVM classifier has the best fault classification performance through the use of the feature reduction methods based on S-LLE. The experimental results indicate that the proposed approach obviously improves the fault classification performance, and can be an effective and efficient tool for rolling bearing fault diagnosis. Therefore, we can safely make use of S-LLE in order to extract the most effective and salient features for classification in practical applications.
